# Long-Term Effectiveness and Outcomes of Bariatric Surgery: A Comprehensive Review of Current Evidence and Emerging Trends

**DOI:** 10.7759/cureus.66500

**Published:** 2024-08-09

**Authors:** Poosarla Ram Sohan, Chandrashekhar Mahakalkar, Shivani Kshirsagar, Shruthi Bikkumalla, Srinivasa Reddy, Akansha Hatewar, Sparsh Dixit

**Affiliations:** 1 General Surgery, Jawaharlal Nehru Medical College, Datta Meghe Institute of Higher Education & Research, Wardha, IND

**Keywords:** long-term outcomes, quality of life, comorbidities, weight loss, bariatric surgery, obesity

## Abstract

Obesity is a global epidemic associated with an increased risk of severe health conditions such as type 2 diabetes, cardiovascular diseases, and certain cancers. Bariatric surgery has become a pivotal treatment for severe obesity, offering significant improvements in weight loss and comorbidity resolution. This comprehensive review aims to assess the long-term effectiveness and outcomes of various bariatric surgical procedures, highlighting current evidence and emerging trends in the field. We extensively reviewed the literature, including randomized controlled trials, cohort studies, and meta-analyses, to evaluate long-term weight loss, resolution of obesity-related comorbidities, quality of life (QoL), and complications associated with different bariatric procedures. Bariatric surgery has demonstrated substantial and sustained weight loss over the long term, with varying degrees of effectiveness among different procedures. Gastric bypass and sleeve gastrectomy are associated with significant improvements in comorbidities such as type 2 diabetes and hypertension. QoL outcomes are generally positive, improving physical health, mental well-being, and social functioning. However, long-term complications, including nutritional deficiencies and the need for reoperations, remain challenges. Emerging trends such as minimally invasive techniques and nonsurgical interventions show promise in enhancing patient outcomes. Bariatric surgery remains a highly effective intervention for managing severe obesity and its related health issues. While long-term outcomes are generally favorable, continued advancements in surgical techniques and postoperative care are crucial for optimizing results and minimizing complications. Future research should focus on personalized approaches to patient management and the development of novel treatment modalities to further improve outcomes in the long term.

## Introduction and background

Obesity is a complex and multifactorial condition characterized by excessive accumulation of body fat, which can adversely affect health [1]. Globally, the prevalence of obesity has risen dramatically over the past few decades, becoming a significant public health concern. The WHO estimates that over 650 million adults are obese, contributing to a significant burden of noncommunicable diseases such as type 2 diabetes, cardiovascular diseases, and certain types of cancer [1]. The etiology of obesity involves a combination of genetic, environmental, and behavioral factors, and its management often requires comprehensive and long-term strategies. Obesity not only reduces life expectancy but also impairs quality of life (QoL), leading to increased morbidity and healthcare costs [2].

Bariatric surgery has emerged as an effective treatment option for severe obesity and its associated comorbidities. The history of bariatric surgery dates back to the 1950s, when jejunoileal bypass was first introduced as a weight-loss procedure. However, due to significant complications and adverse effects, this approach was mainly abandoned [3]. The subsequent development of gastric bypass, pioneered by Dr. Edward E. Mason in the 1960s, marked a significant advancement in the field. Since then, bariatric surgery has evolved with various procedures such as vertical banded gastroplasty, adjustable gastric banding, sleeve gastrectomy, and biliopancreatic diversion with a duodenal switch. These procedures have demonstrated substantial improvements in weight loss and the resolution of obesity-related comorbidities [3].

This comprehensive review aims to evaluate bariatric surgery’s long-term effectiveness and outcomes, focusing on current evidence and emerging trends. The review will explore various aspects, including sustained weight loss, improvement in obesity-related comorbidities, QoL, and potential complications. Additionally, it will discuss the evolution of surgical techniques, advancements in postoperative care, and emerging trends in the field. By synthesizing existing literature and recent studies, this review seeks to provide a holistic understanding of bariatric surgery’s role in managing obesity and its long-term implications.

## Review

Types of bariatric surgery

**Figure 1:**
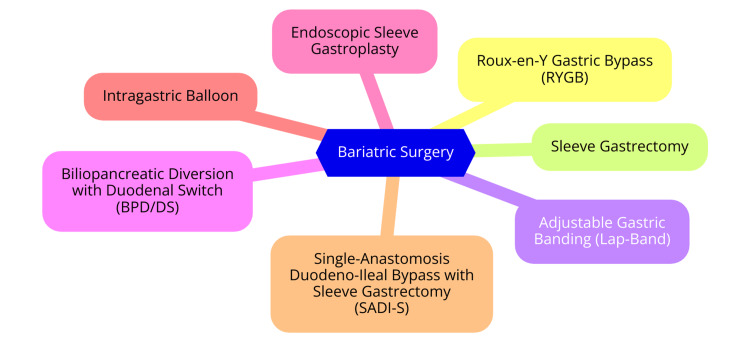
Types of bariatric surgery Image credit: Poosarla Ram Sohan

Bariatric surgery includes a range of surgical procedures designed to facilitate weight loss and improve health conditions related to obesity. One of the most prevalent bariatric surgeries is the Roux-en-Y gastric bypass (RYGB). This procedure involves creating a small pouch at the top of the stomach, which limits food intake, and rerouting the small intestine to this pouch. RYGB restricts food consumption and reduces calorie and nutrient absorption by bypassing a portion of the small intestine. It has been proven effective in promoting significant weight loss and improving obesity-related conditions, including type 2 diabetes [4]. Sleeve gastrectomy entails the removal of approximately 80% of the stomach, leaving a tubular structure that resembles a banana. This procedure dramatically reduces the stomach’s capacity, decreasing food intake. Additionally, it lowers the production of ghrelin, the hunger hormone, which can reduce appetite. While effective for weight loss, sleeve gastrectomy does not affect nutrient absorption in the intestines [5]. Adjustable gastric banding, commonly known as the Lap-Band, involves placing a silicone band around the upper part of the stomach to create a small pouch. This restricts food intake and can be adjusted postoperatively to alter the size of the pouch. Considered the least invasive of the bariatric procedures, it is typically recommended for patients with less weight to lose. However, it is generally less effective than other procedures for achieving long-term weight loss [6]. Biliopancreatic diversion with a duodenal switch is a complex procedure that combines both restrictive and malabsorptive techniques. It involves removing a significant portion of the stomach and rerouting the intestines to limit food intake and nutrient absorption. This procedure is effective for substantial weight loss and is often reserved for patients with severe obesity or those who have not achieved the desired results with other surgeries. However, it carries a higher risk of nutritional deficiencies and complications [7]. Recent advancements in bariatric surgery include various modifications and new techniques to improve outcomes and reduce complications. These innovations may involve less invasive approaches, such as robotic-assisted surgeries, and the development of hybrid procedures that combine elements of existing surgeries. Ongoing research is focused on evaluating the efficacy and safety of these emerging techniques and their long-term impacts on weight loss and metabolic health [8]. Types of bariatric surgery are shown in Figure 1.

Mechanisms of action

Bariatric surgery employs various mechanisms to achieve weight loss and metabolic improvements, primarily through restrictive and malabsorptive effects, hormonal changes, and impacts on the gut microbiota [9]. Restrictive procedures, such as sleeve gastrectomy, focus on reducing the stomach size and limiting the amount of food consumed at one time. This reduction leads to early satiety, where patients feel full after consuming smaller meals. A smaller stomach size promotes quicker gastric filling, inducing feelings of fullness and thus reducing overall caloric intake [10]. Conversely, malabsorptive procedures, like RYGB, restrict food intake and alter the digestive process by bypassing a significant portion of the small intestine. This modification reduces nutrient absorption by limiting the area where nutrients are absorbed into the bloodstream. These restrictive and malabsorptive mechanisms contribute to weight loss and metabolic changes, including improved glucose metabolism [11]. In addition to physical changes, bariatric surgery triggers significant hormonal changes crucial for weight loss and metabolic health. Post-surgery, there is a notable increase in the secretion of gut hormones such as glucagon-like peptide-1 (GLP-1) and peptide YY (PYY) [12]. These hormones play critical roles in appetite regulation and insulin sensitivity. Elevated levels of GLP-1 and PYY contribute to reduced appetite and enhanced glucose tolerance, promoting weight loss and potentially leading to the remission of type 2 diabetes. Moreover, these hormonal changes improve insulin dynamics, boosting insulin sensitivity and secretion. This is particularly beneficial for patients with obesity-related type 2 diabetes, often resulting in better glycemic control and, in many cases, remission of the condition [12]. Emerging evidence also suggests that bariatric surgery impacts gut microbiota composition, which may further influence metabolic outcomes [13]. Post-surgery changes in the gut microbiome can enhance metabolic health by improving nutrient absorption and modulating inflammation. Specific microbial profiles have been linked to better insulin sensitivity and reduced obesity-related complications. This interaction between bariatric surgery and the gut microbiota highlights the complexity of the mechanisms involved in weight loss and metabolic improvement [13]. The role of various medications in conjunction with bariatric surgery for weight management and metabolic improvement is shown in Table 1.

**Table 1:** The role of various medications in conjunction with bariatric surgery for weight management and metabolic improvement

Medication	Class	Mechanism of action	Efficacy	Integration with bariatric surgery
Liraglutide (Saxenda)	GLP1 agonist	Mimics GLP1, increasing insulin secretion and reducing appetite	Significant weight loss; reduces HbA1c	Often used pre- and postoperatively to enhance weight loss and improve glycemic control
Semaglutide (Ozempic)	GLP1 agonist	Increases insulin secretion, decreases glucagon, and reduces appetite	High efficacy in weight loss; lowers HbA1c levels	Can be used in combination with surgical procedures to boost weight loss outcomes and metabolic health
Lorcaserin (Belviq)	Serotonin 2C receptor agonist	Suppresses appetite by activating serotonin receptors in the brain	Modest weight loss	Limited integration due to concerns about long-term safety
Phentermine/topiramate (Qsymia)	Sympathomimetic/anticonvulsant	Reduces appetite and increases energy expenditure	Significant weight loss	Often used as a short-term adjunct to surgical interventions
Naltrexone/bupropion (Contrave)	Opioid antagonist/antidepressant	Modulates the hypothalamus and mesolimbic dopamine circuit	Moderate weight loss	Used to manage weight regain post-surgery
Orlistat (Xenical)	Lipase inhibitor	Inhibits fat absorption in the intestines	Modest weight loss; reduces fat absorption	May be used post-surgery to help maintain weight loss, but gastrointestinal side effects can be a limitation
Metformin	Biguanide	Reduces hepatic glucose production and improves insulin sensitivity	Modest weight loss; improves glycemic control	Commonly used in patients with type 2 diabetes undergoing bariatric surgery to improve metabolic outcomes
Canagliflozin (Invokana)	SGLT2 inhibitor	Increases glucose excretion in the urine	Modest weight loss; lowers blood sugar levels	Can be used in combination with surgical procedures to improve glycemic control in patients with type 2 diabetes
Setmelanotide	MC4R agonist	Targets MC4Rs to regulate appetite and energy expenditure	Effective for genetic obesity syndromes	Primarily used in specific genetic conditions but may have future implications for broader bariatric surgery applications
Pramlintide (Symlin)	Amylin analog	Slows gastric emptying, suppresses glucagon, and reduces appetite	Modest weight loss; improves postprandial glucose control	Can be used to manage postsurgical hyperglycemia and support additional weight loss

Long-term weight-loss outcomes

Bariatric surgery has proven to be the most effective long-term treatment for severe obesity, leading to significant weight loss and improvements in obesity-related comorbidities that can persist for many years. Studies show that patients typically maintain a substantial portion of their weight loss over time, although outcomes can vary depending on the procedure [14]. Research indicates that patients can sustain their weight loss for extended periods of time. Five years post-surgery, individuals often maintain an average reduction of approximately 23.4% of their initial weight. This trend continues at the 10-year mark, with patients who underwent RYGB retaining 50-60% of their excess weight loss. By the 15-year follow-up, patients generally experience an average weight reduction of 22.2% of their initial body weight, corresponding to a sustained loss of around 30.1 kg. These statistics underscore the durability of weight loss achieved through bariatric surgery [15]. Different surgical procedures yield varying outcomes. Gastric bypass typically results in an average weight loss of about 70% of the excess body weight. At the same time, the duodenal switch procedure often leads to even more significant weight loss, averaging around 80% of excess weight. Sleeve gastrectomy shows a broader range of outcomes, with weight loss generally falling between 30% and 80% of excess weight within 18-24 months postoperatively. Notably, a randomized trial found that patients who underwent gastric bypass experienced more significant improvement in type 2 diabetes at the three-year follow-up compared to those who had sleeve gastrectomy, suggesting that the choice of procedure can affect long-term health outcomes [16]. Several factors are critical for long-term weight loss success following bariatric surgery. Achieving an initial weight loss of at least 20 kg during the preoperative program is strongly associated with better long-term maintenance. Additionally, lower levels of disinhibited eating and food addiction at the end of the weight-loss intervention have been linked to successful maintenance at the two-year mark. Regular physical activity is also a significant predictor of long-term success; individuals who engage in physical activity equivalent to walking approximately 28 miles per week are more likely to maintain their weight loss [17].

Long-term health outcomes

Bariatric surgery offers significant long-term health benefits, particularly in resolving or improving comorbidities, enhancing cardiovascular health, and extending overall mortality and life expectancy. Numerous studies highlight the profound impact of bariatric surgery on obesity-related conditions. For instance, many patients experience complete remission or substantial improvement in glycemic control after surgery. A meta-analysis indicates that up to 80% of patients with type 2 diabetes achieve remission within two years of surgery, with these benefits often sustained over the long term [18]. Research also demonstrates that bariatric surgery can lead to reduced blood pressure levels, with many patients able to discontinue antihypertensive medications postoperatively. Nearly 50% of hypertensive patients achieve normal blood pressure following the procedure. Additionally, improvements in lipid profiles are expected, including reductions in total cholesterol, low-density lipoprotein cholesterol, and triglycerides, along with increases in high-density lipoprotein cholesterol levels. These changes contribute to enhanced cardiovascular health [19]. Bariatric surgery is associated with a decreased incidence of cardiovascular events. Observational studies reveal that patients undergoing metabolic or bariatric surgery experience a reduced risk of heart disease and related complications. Specifically, the surgery is linked to lower rates of myocardial infarction and stroke, improved heart function, and reduced left ventricular mass in obese patients with heart failure. These cardiovascular benefits are attributed to substantial weight loss and the resolution of comorbidities such as diabetes and hypertension [20]. The long-term effects of bariatric surgery also extend to overall mortality and life expectancy. Studies show that patients who undergo bariatric surgery have a significantly lower risk of death from all causes compared to those who do not have the surgery [21]. A systematic review found that bariatric surgery is associated with a 29% reduction in all-cause mortality over a follow-up period exceeding 10 years. Furthermore, patients often experience improvements in life expectancy, with some studies suggesting an increase of up to five to 10 years post-surgery, primarily due to reductions in obesity-related complications and enhanced metabolic health [22].

QoL and psychological outcomes

Bariatric surgery significantly enhances the QoL and psychological well-being of patients with obesity. Research consistently shows that individuals undergoing these procedures experience notable improvements in various aspects of their lives. Studies using standardized questionnaires, such as the SF-36, reveal that patients report better physical and mental health following surgery [23]. For instance, one cohort study observed QoL scores increasing from a baseline of 48.3-79.7 after one year and to 65.1 after 10 years, indicating sustained benefits over time. Patients frequently report improvements in physical functioning, mobility, and overall health status, along with substantial reductions in depression and anxiety. A long-term study found that surgical patients experienced better perceived health and mood, emphasizing the psychological benefits associated with weight loss [24]. The long-term effects of bariatric surgery also extend to body image and self-esteem. As patients lose weight, many experience a shift toward a more positive self-perception, leading to increased self-confidence and greater social engagement. Enhanced satisfaction with appearance is commonly reported and correlates with weight loss and improved health outcomes [25]. Research suggests that the psychological benefits of improved body image can be as significant as physical health improvements. Enhanced self-esteem is a frequent outcome, with many patients feeling more comfortable in social situations and more active in their daily lives. This transformation is often linked to reducing the stigma associated with obesity, allowing individuals to embrace new opportunities [26]. The social and economic implications of these changes are also substantial. Patients often report improved social interactions and relationships, leading to better support networks. Economically, the benefits are significant; many patients return to work or enhance their job performance due to increased energy levels and confidence. The reduction in obesity-related comorbidities also results in lower healthcare costs over time. Studies suggest that the cost-effectiveness of bariatric surgery is favorable, especially when considering the long-term savings associated with improved health outcomes and reduced medical interventions [27].

Complications and risks

Bariatric surgery, while effective in achieving weight loss and improving obesity-related health conditions, carries a range of complications and risks that patients should be aware of. These complications can be categorized into immediate postoperative issues, long-term complications, and the potential need for reoperations or revisions [28]. Immediate postoperative complications may arise shortly after the surgery and include infections, hemorrhage, and anesthesia-related issues. Surgical site infections can delay healing and increase morbidity, while excessive bleeding during or after surgery may necessitate transfusions or additional interventions [29]. Reactions to anesthesia also pose risks, particularly for patients with preexisting comorbid conditions. Additionally, thromboembolic events, such as deep vein thrombosis or pulmonary embolism, can occur due to immobility during the postoperative period. Procedures like gastric bypass and sleeve gastrectomy also carry the risk of leakage from the staple line, potentially leading to severe complications such as peritonitis [30]. Long-term complications can develop months or even years after surgery and often require ongoing management. A significant concern is nutritional deficiencies resulting from altered digestion and absorption. Patients may experience deficiencies in essential nutrients such as vitamin B12, iron, calcium, and vitamin D, potentially leading to health issues like anemia and osteoporosis. Lifelong monitoring and supplementation are necessary to address these deficiencies [31]. Gastrointestinal issues are also common, with conditions such as dumping syndrome, which causes food to move too quickly from the stomach to the small intestine, leading to symptoms like nausea and diarrhea. Other complications may include gastroesophageal reflux disease, internal hernias, and marginal ulcers, all of which can significantly affect a patient’s QoL [32]. Reoperations and revisions may be necessary for some patients, particularly if they experience weight regain or complications from the initial surgery. Studies suggest that approximately 10-20% of patients may require a revision within five to 10 years post-surgery. Reasons for reoperations can include issues such as leaks, strictures, or internal hernias that require surgical intervention. In procedures involving adjustable gastric bands, complications like band slippage or erosion may necessitate the removal or replacement of the device [33].

Emerging trends and future directions

Bariatric surgery is evolving rapidly, marked by significant advancements in surgical techniques, nonsurgical alternatives, personalized medicine, and postoperative care. These emerging trends are shaping the future of obesity treatment, improving patient outcomes, and expanding access to care [34]. A prominent trend in bariatric surgery is the shift toward minimally invasive surgical techniques. Laparoscopic and robotic-assisted surgeries have revolutionized traditional approaches by offering smaller incisions, reduced scarring, and faster recovery times. Robotic systems, such as the da Vinci Surgical System, enhance precision and control, allowing surgeons to perform complex procedures with improved dexterity and visualization [35]. Additionally, augmented reality and virtual reality are being incorporated into surgical training and planning, enabling surgeons to practice procedures in a virtual environment and providing real-time data during surgeries. Innovations like three-dimensional printing are also used to create patient-specific models and implants, improving surgical outcomes through better preoperative planning [36]. In parallel with surgical advancements, nonsurgical alternatives are gaining prominence in obesity management. Endoscopic procedures, such as sleeve gastroplasty, are emerging as effective, less invasive options than traditional bariatric surgery [37]. These procedures can often be performed without general anesthesia, making them accessible to a broader range of patients. Additionally, new anti-obesity medications are being developed, providing further options for weight management. These pharmacotherapies can complement surgical interventions or serve as standalone treatments for patients who are not candidates for surgery, thereby broadening the scope of obesity management [37]. The future of bariatric surgery is also leaning toward personalized medicine, where treatment plans are customized based on individual patient characteristics, including genetic profiles. Understanding genetic predispositions to obesity can assist healthcare providers in selecting the most effective surgical or nonsurgical treatment options for each patient. This personalized approach aims to enhance outcomes and minimize complications by ensuring patients receive interventions tailored to their needs [38]. Innovations are also transforming postoperative care and follow-up strategies in bariatric surgery. The integration of telemedicine facilitates improved monitoring and follow-up of patients after surgery, enhancing patient engagement and adherence to follow-up protocols, which are crucial for long-term success [39]. Wearable technology is increasingly popular, allowing patients to track their activity, dietary habits, and vital signs in real time. This data can be shared with healthcare providers, enabling timely interventions and personalized support. Furthermore, Enhanced Recovery After Surgery protocols are being adopted to optimize postoperative recovery through better pain management, nutrition, and mobilization strategies, ultimately reducing hospital stays and improving overall recovery times [39]. Current evidence and emerging trends in bariatric surgery are shown in Table 2.

**Table 2:** Current evidence and emerging trends in bariatric surgery 3D, three-dimensional; AR, augmented reality; ERAS, Enhanced Recovery After Surgery; ESG, endoscopic sleeve gastroplasty; NIH, National Institutes of Health; QoL, quality of life; RYGB, Roux-en-Y Gastric bypass; SADIS, sleeve gastrectomy with duodenal switch; SAGI, sleeve gastrectomy with ileal interposition; VR, virtual reality

Category	Current evidence	Emerging trends
Surgical techniques	RYGB: effective for long-term weight loss and comorbidity resolution	Robotic-assisted surgeries: increased precision and reduced recovery times
	Sleeve gastrectomy: high efficacy in weight loss and reduction in hunger hormones	AR and VR: enhanced surgical training and planning
	Adjustable gastric banding: less invasive but lower long-term weight loss outcomes	Hybrid procedures: combining elements of different surgeries for better outcomes
	Biliopancreatic diversion with duodenal switch: effective but higher risk of complications	3D printing: custom models and implants for patient-specific procedures
Endoscopic procedures	ESG: minimally invasive with promising weight loss results	Endo Barrier Gastrointestinal Liner: a nonsurgical option showing effective weight loss and metabolic improvements
	Intragastric balloons: temporary and minimally invasive, but with variable efficacy	Incisionless anastomosis systems: minimizing invasiveness and recovery time
Combination procedures	SADIS: effective for severe obesity	SAGI: improved weight loss and metabolic outcomes
	Gastric bypass with transit bipartition: effective but requires more research	Sleeve gastrectomy with transit bipartition: combining restrictive and malabsorptive elements for better efficacy
Pharmacotherapy	GLP1 agonists (liraglutide and semaglutide): effective adjuncts to surgical procedures	New anti-obesity medications: developing drugs that target various aspects of appetite and metabolism
	Metformin: commonly used in type 2 diabetes patients undergoing surgery	Combination therapies: using multiple medications for synergistic effects
	Orlistat: limited by gastrointestinal side effects	Personalized medicine: tailoring drug therapy based on genetic profiles and individual characteristics
Postoperative care	Lifelong nutritional supplementation: essential to prevent deficiencies	Telemedicine: enhanced monitoring and follow-up through digital health platforms
	Regular monitoring for complications: critical for long-term success	Wearable technology: real-time tracking of physical activity, diet, and vital signs
	Psychological support: important for maintaining long-term weight loss	ERAS protocols: improved postoperative recovery and reduced hospital stays
Patient selection	NIH criteria for candidacy: BMI and comorbidity guidelines	Advanced diagnostic tools: using biomarkers and imaging to better select and prepare patients
	Preoperative evaluation: comprehensive medical, nutritional, and psychological assessment	Genetic testing: identifying patients with genetic predispositions to better tailor surgical and medical interventions
QoL outcomes	Improved physical and mental health: significant improvements post-surgery	Long-term studies: ongoing research into the durability of QoL improvements
	Enhanced self-esteem and body image: common among postsurgical patients	Integration of mental health support: more structured and continuous psychological support post-surgery

Patient selection and preoperative considerations

Selecting appropriate candidates for bariatric surgery is crucial for achieving successful outcomes. The National Institutes of Health has established criteria for surgical candidacy, which have evolved. Critical criteria include a BMI of 40 or higher or a BMI of 35 or higher with obesity-related comorbidities (such as type 2 diabetes or hypertension) [40]. Candidates should have previously attempted and failed nonsurgical weight loss methods, such as dietary changes and exercise programs. Psychological stability and readiness for post-surgery lifestyle changes are also essential, often requiring a multidisciplinary team approach to ensure a comprehensive evaluation. While most candidates are between 18 and 65, exceptions can be made based on individual circumstances. The presence of obesity-related health issues can impact candidacy, as successful surgery may lead to significant improvements in these conditions [40]. A thorough preoperative evaluation is critical for optimizing surgical outcomes. This evaluation typically includes a comprehensive review of the patient’s medical history, including any existing comorbidities. Nutritional assessments evaluate dietary habits and nutritional status to identify and address any deficiencies before surgery. Psychological assessments screen for mental health conditions and ensure that patients understand the implications of surgery and the necessary lifestyle changes [41]. Collaboration among surgeons, dietitians, psychologists, and other healthcare professionals is essential to tailor the approach to each patient’s needs. Patients must be fully informed about the surgery’s risks, benefits, expected outcomes, and the commitment required for long-term success [41]. Several factors can predict the success of bariatric surgery. Regular follow-up appointments to monitor weight loss, nutritional status, and psychological well-being are crucial for long-term success [42]. A strong commitment to lifestyle changes, including diet and exercise, significantly impacts weight loss and overall health improvements post-surgery. The choice of surgical procedure may affect outcomes, with RYGB often leading to more significant weight loss compared to adjustable gastric banding. Patients who achieve some weight loss before surgery may experience better outcomes, as this can demonstrate their commitment to the process and help reduce surgical risks. A robust support system, including family, friends, and support groups, can enhance motivation and adherence to post-surgical guidelines [42].

Postoperative management and follow-up

Effective postoperative management and follow-up care are essential for the long-term success of bariatric surgery. This comprehensive approach encompasses nutritional management, ongoing monitoring, lifestyle modifications, and the collaborative efforts of a multidisciplinary care team [43]. Post-bariatric surgery patients encounter unique nutritional challenges due to altered digestion and absorption. A specific dietary regimen is recommended to address these challenges, progressing from liquids to solid foods over several weeks. This diet typically emphasizes small portion sizes, high protein intake, and low-fat foods to support healing and promote weight loss [44]. Lifelong supplementation is also necessary to prevent deficiencies in essential vitamins and minerals, with common recommendations including multivitamins, iron, calcium, and vitamin B12. Regular monitoring of nutritional status is critical, with blood tests usually conducted at six months, 12 months, and annually after that to adjust supplementation as needed [44]. Ongoing monitoring is crucial for assessing patients’ physical and psychological health post-surgery. Patients should have at least annual follow-up appointments with their healthcare provider to review their weight, nutritional status, and any comorbidities [45]. These visits should also include assessments for potential complications, such as dumping syndrome or nutritional deficiencies. Additionally, patients are encouraged to maintain a healthy lifestyle that includes regular physical activity and behavioral modifications. This may involve setting realistic goals, tracking food intake, and participating in support groups to maintain motivation and accountability [45]. The complexity of postoperative care necessitates a multidisciplinary approach. Key components of this team include nutritional therapists, psychologists, and primary care providers. Nutritional therapists offer guidance on dietary changes and supplementation needs and help patients navigate the challenges of post-surgery eating habits [46]. Mental health professionals address emotional and psychological issues that may arise after surgery, such as body image concerns and emotional eating. They also assist in developing coping strategies for lifestyle changes. Primary care providers play a crucial role in the long-term management of bariatric patients, ensuring appropriate monitoring and referrals to specialists as needed. They also help manage chronic conditions that may persist or emerge post-surgery [46].

## Conclusions

Bariatric surgery represents a transformative intervention for managing severe obesity and its related health complications. Patients have demonstrated substantial and sustained weight loss through various surgical approaches, improved resolution of obesity-related comorbidities, and enhanced QoL. Despite the significant benefits, long-term outcomes are influenced by factors such as the type of surgery, patient adherence to lifestyle changes, and effective postoperative management. As the field advances with new surgical techniques, adjunctive therapies, and personalized care strategies, ongoing research is essential to refine practices, minimize risks, and optimize patient outcomes. This comprehensive review underscores the importance of integrating evidence-based approaches with emerging trends to ensure bariatric surgery’s continued effectiveness and safety in the fight against obesity.
